# Cross-cultural adaptation and psychometric properties of the Herth Hope Index in Kinyarwanda: adapting a positive psychosocial tool for healthcare recipients and providers in the Rwandan setting

**DOI:** 10.1186/s12955-020-01537-3

**Published:** 2020-08-24

**Authors:** Angele Bienvenue Ishimwe, Julia Kaufman, Delphine Uwamahoro, Jonathan Taylor Wall, Kaye Herth, Emery Chang, Jean de Dieu Ngirabega, Wendy Leonard

**Affiliations:** 1 TIP Global Health, PO Box 1285, KN 4 St, Kigali, Rwanda; 2 Duke Global Health Institute, Duke University, 310 Trent Dr, Durham, NC 27710 USA; 3 Minnesota State University, Mankato, 228 Wiecking Center, Mankato, MN 56001 USA; 4 UCLA Health, 757 Westwood Plaza, Los Angeles, CA 90095 USA; 5 East African Health Research Commission, Quartier Kigobe, Avenue des Etats Unis, no. 71, B.P, 350 Bujumbura, Burundi

**Keywords:** Hope, Cross-cultural, Positive instrument development, Psychometrics, Frontline healthcare

## Abstract

**Background:**

The lack of culturally appropriate instruments to measure hope across cultural settings is a barrier to assessing and addressing the relationship between hope and health outcomes. The study aim was to adapt and evaluate the psychometric properties of the Herth Hope Index (HHI) in Kinyarwanda in a population of healthcare recipients and healthcare workers in Rwanda.

**Methods:**

A transcultural translation and adaptation of the HHI was conducted using qualitative methods (*n* = 43) to achieve semantic, content, and technical equivalence. The adapted instrument was administered to a purposive sample (*n* = 206) of Rwandan healthcare patients and providers. Temporal reliability, internal reliability using Cronbach’s alpha, and construct validity using confirmatory factor analysis (CFA) were assessed.

**Results:**

The Herth Hope Index-Kinyarwanda (HHI-K) was found to have strong internal consistency (α = 0.85) and test-retest reliability (*r* = 0.85). The original HHI three-factor structure fit the data well in CFA (normed chi-square = 1.53; root mean square error of approximation = 0.05; standardized root mean square residual = 0.05; comparative fit index = 0.96; Tucker-Lewis Index = 0.95).

**Conclusion:**

This article presents the first rigorous cultural adaptation of the HHI in a low-income country. The HHI-K has acceptable psychometric properties, resulting in a new useful tool for research, program development, and evaluation in Rwandan healthcare settings. The HHI-K instrument can be used to assess the effectiveness of programs that aim to promote hope and health outcomes across health system- and individual-levels. The process also provides a feasible model for adaptation of a positive psychosocial tool for both patients and providers in low-resource settings.

## Background

Hope is the desire for and belief in a positive future, defined as “a multidimensional, dynamic, empowering state of being that is central to life, related to external help and caring, oriented towards the future and highly personalized to each individual.” [1] Hope is theorized as motivating individuals to act towards reaching a future goal, manifesting emotionally and behaviorally, and relating to both abstract future expectations and expectations of concrete outcomes [2, 3].

Hope has been conceptualized as a positive psychosocial strength influenced by the social environment while protecting against risk, making it relevant to the context of healthcare [4]. Hope is also considered an important mediator between risk factors in low-resource settings and engagement in risky health behaviors [5]. While long-term illnesses like HIV can induce hopelessness, research has shown that modest interventions that instill a sense of hope can lead to improvements in related health and education outcomes [6, 7]. Hope has been measured in relation to a variety of physical and psychological factors, including pain, fatigue, psychological distress, quality of life, and family support [8–10]. Although a lack of hope is associated with depression and anxiety, hope as a multidimensional concept represents more than the absence of clinically-relevant depressive and anxious symptoms [11, 12]. Loss of hope has been correlated with lower functioning in both physical and psychological domains [13–15]. Hope is therefore a key indicator for understanding patient wellbeing and an important target for intervention outcomes.

Hope has increasingly been shown have significant importance to healthcare workers as well. The wellbeing of healthcare workers is critical to the effective functioning of healthcare systems. High levels of stress can negatively affect the wellbeing of healthcare workers and lead to burnout, in which they experience emotional exhaustion, depersonalization from patients, and reduced personal accomplishment [16, 17]. For example, a study conducted in Zimbabwe considered the motivations and frustrations among healthcare workers after the rollout of antiretrovirals for HIV-positive individuals. Although access to antiretrovirals initially improved optimism among healthcare workers, the intermittent shortages of medications mitigated the initial increase [18]. Another study in Malawi found that both individual-level vulnerabilities along with work-related modifiable factors, such as the lack of a supportive supervisor and dissatisfaction with team interactions, are associated with higher rates of burnout [19]. To better understand and address the role of hope related to health-promoting behaviors among patients and service provision by frontline health workers, valid and reliable instruments to measure hope in a variety of cultural settings are needed.

Instruments to measure hope have mainly been developed in high-income countries and in clinical settings [20, 21]. One commonly used instrument to measure hope is the Herth Hope Scale (HHS) and its abbreviated version, the Herth Hope Index (HHI) [22, 23]. The HHI is a 12-item instrument designed to measure hope based on the multidimensional concept of hope as theorized by Dufault and Martocchio. The three dimensions are 1) inner sense of temporality and future, 2) inner positive readiness and expectancy, and 3) interconnectedness with self and others [23]. The concurrent criterion-related validity of HHI to HHS is high (0.92). The HHI maintained the three-factor structure of the HHS, which accounted for 61% of the total variance [23]. The answers are given on a 4-point Likert-type scale (1 = strongly disagree to 4 = strongly agree). The score is calculated by summing each item’s score, with items #3 and #6 reverse-scored. Scores range from 4 to 48, and a higher value signifies higher hope. HHI items #2 and #4 were reworded in 1999 to make their meanings clearer [24]. In a sample of 172 adult patients, its psychometric features were excellent, with a Cronbach’s alpha of 0.97 and a test-retest reliability after 2 weeks of 0.91 [23]. Concurrent validity with other scales ranged from 0.92 with the Nowotny Hope Scale, which measures hope among cancer patients, to − 0.73 with Beck’s Hopelessness Scale, which measures suicidal tendency [25, 26].

The HHI has been widely used in research and clinical practice in different cultural settings for various health areas [27]. Translations of the HHI into Chinese [28], Dutch [29], German [30], Italian [31], Japanese [32], Norwegian [33], Persian [34], Portuguese [35], Spanish [36], and Swedish [37] have been published with satisfactory psychometric properties.

No translation or cross-cultural evaluation of the HHI in low- and middle-income countries (LMIC) has yet been published. Given the importance of hopefulness to poverty alleviation and health interventions, reliable and validated instruments to measure hope across cultural contexts are needed to better understand and address the relationship between hope and health.

### Aims

The aims of this study were to transculturally adapt the Herth Hope Index to Kinyarwanda and evaluate its psychometric properties, specifically examining reliability and construct validity. The study also sought to demonstrate the feasibility of cross-culturally adapting and validating a positive psychological measure simultaneously for healthcare workers and recipients and to serve as a model for such adaptations in low-resource settings.

## Methods

### Setting

Rwanda, has achieved major development gains in the last decade with widespread health improvements among its 12 million people [38, 39]. However, in part due to the historical context of the 1994 genocide and its lasting effects, hope and mental health are important considerations when creating, implementing, and evaluating health programs [40, 41]. Depression, anxiety, and post-traumatic stress disorder are prevalent in Rwanda, with rates twice as high in women compared with men [42]. Further, Rwanda’s community-based health insurance scheme covers over 81.6% of the population and continues to grow—a successful step towards universal health coverage (UHC) and the Sustainable Development Goals [43]. As health coverage expands, frontline healthcare workers must provide a growing number of services to an increasing proportion of the population. A well-supported, trained, equipped, and paid health workforce—linked to hopefulness—will better enable the country’s efforts to achieve UHC [44].

The research took place in the Ruli District Hospital catchment area, located in Gakenke District in the Northern Province of Rwanda, which includes nine health centers and serves about 130,000 individuals. In Ruli Catchment area in 2018, there were 54 maternal and child healthcare workers, 144 total healthcare workers, and 3308 women newly enrolled in antenatal care. The research was conducted by TIP Global Health (TIP), a Rwandan non-governmental community health organization based in Ruli, Rwanda. TIP works with pregnant women and mothers, many of whom are living with HIV, as well as other healthcare recipients and their healthcare workers to create community-driven health programs that strengthen national health systems. TIP plans to develop and implement a program to promote hope for the healthcare workers and recipients who are supported by TIP’s interventions; TIP conducted this research to better understand and address the critical influence of hope among healthcare workers and recipients, including assessing the impact and effectiveness of relevant interventions.

### Transcultural translation and adaptation

A systematic approach for transcultural translation and adaptation was developed in Nepal and has been widely used as part of cross-cultural mental health studies [45–47]. The approach uses qualitative methods to optimize semantic, technical, content, and conceptual equivalence of a culturally adapted tool with the original tool. The process to transculturally translate and adapt the HHI to Kinyarwanda, creating the Herth Hope Index – Kinyarwanda (HHI-K), was derived from the tool developed by van Ommeren and colleagues and involved three main steps [45]. A team of five bilingual translators at TIP with extensive experience in rural Rwandan health settings created the first draft of the Kinyarwanda tool. Next, a series of focus group discussions and cognitive interviews were conducted by the research team with healthcare workers and healthcare recipients. Adjustments were made based on the feedback of participants. Lastly, two blind back-translations were completed. Both the pre- and post-translation English versions were reviewed, and final modifications were made to the instrument to meet the original conceptual objectives. The approach optimized equivalence by evaluating four criteria at each step using a transcultural monitoring form: comprehensibility, acceptability, relevance, and completeness [﻿41﻿, ﻿44﻿].

1. Comprehensibility of an item is a measure of semantic equivalence and relates to using appropriate terminology, such as idioms of distress [48]. If an item is deemed comprehensible by a focus group or individual, it is assumed to be understandable by a general audience in the local context [49].
2. Acceptability reflects that an item is not overly sensitive for people to endorse (e.g., items about sex or alcohol). This section also considers technical equivalence in how data are collected across cultures. Likert scales in particular require assessment to determine if they are understood across cultures. If an item is deemed to have an acceptable response set, it suggests that respondents will rate items similarly to the original intention of the instrument [49].
3. Relevance pertains to content equivalence. If an item is relevant, it has significant meaning in the cultural setting [49]. For example, difficulty concentrating on television or distracted driving are relevant to symptoms of depression in some cultural settings but not others.
4. Completeness, which addresses semantic and conceptual equivalence, refers to whether a question relates to the same concepts as the original item. Completeness accounts for cultural norms in relation to markers of distress [49]. In this study, completeness refers to whether the item reflects the experience of hopefulness.

### Participants for transcultural translation

Participants for the focus group discussions, which included both healthcare recipients and workers, were purposively sampled from nine community health centers in the Ruli catchment area. Participants were selected according to their alignment with the population TIP works with through its community health programs and resembles the anticipated population of TIP’s future intervention to promote hope.

### Participants for psychometric evaluation

For the psychometric evaluation, participants, including both healthcare recipients and healthcare workers, were purposively sampled from nine community health centers in the Ruli District Hospital catchment area. Participants were eligible if they were older than 18 years of age, spoke Kinyarwanda, could complete the consent process, and did not have any issues preventing them from hearing or responding to questions read to them.

### Psychometric evaluation

Reliability is the capacity of an instrument to produce consistent results internally (across items) and in different situations. Reliability was measured with internal consistency to assess the general agreement between all items in the instrument [50]. To measure the internal consistency of the HHI-K, Cronbach’s alpha was calculated [51, 52]. Cronbach’s alpha is a measure of internal consistency based on the degree of inter-correlation among all items on a scale. Internal consistency estimates of .70 or higher are considered sufficient [50]. Test-retest reliability at 1 week (*n* = 48) was assessed by the same survey enumerators to measure the reliability of the instrument across time. The intraclass correlation coefficient (ICC) was used to estimate the test-retest reliability coefficient; estimates above 0.70 are considered acceptable [53].

Confirmatory factor analysis (CFA), using the estimator of maximum likelihood, was used to verify the construct validity of the instrument by determining whether the original factor structure of the HHI generalizes to the HHI-K. Construct validity refers to the degree to which a tool measures what it claims to be measuring [54]. The following indices were examined to assess the goodness-of-fit between the original model and the study sample data: the normed chi square (chi-square index divided by the degrees of freedom), the root mean square error of approximation (RMSEA), the standardized root mean square residual (SRMR), the comparative fit index (CFI), and the Tucker-Lewis Index (TLI). The following criteria were used for evaluation: normed chi-square < 5.0 and preferably < 2.0, RMSEA < 0.08, SRMR < 0.08, CFI > 0.90, and TLI > 0.90. These indices demonstrate whether the model shows a good fit to the data [55, 56]. Statistical analyses were performed using R version 3.5.3, with lavaan version 0.6–3 for CFA modeling.

## Results

### Transcultural translation and adaptation

After completion of the translation by bilingual speakers, the draft of the HHI-K was reviewed by nine focus groups with healthcare recipients, and six focus groups and two interviews with healthcare workers (Table 1). The age of respondents ranged from 22 to 51 years old with a mean of 34. Almost all participants were female (98%), as expected given the target population. Most were married (72%), though legally married and cohabitating participants were not disaggregated for this phase of the research.

**Table 1:** Demographics of focus groups and interviews

Role in Health Center	Total Sample (***n*** = 43)
Healthcare Service User	28 (65%)
Healthcare Worker	15 (35%)
**Age, Mean (Range)**	34 (22–51)
**Gender**	
Male	1 (2%)
Female	42 (98%)
**Ubudehe Category**^a^	
1	5 (12%)
2	14 (33%)
3	24 (55%)
4	0 (0%)
**Education**	
No schooling	1 (2%)
Primary	20 (47%)
Vocational Training School	0 (0%)
Secondary	10 (23%)
Advanced diploma	7 (16%)
Bachelor’s degree	5 (12%)
**Marital Status**	
Single	9 (21%)
Married	31 (72%)
Widowed	1 (2%)
Celibate / Religious	2 (5%)

^a^Ubudehe category refers to the economic classification of individuals based on their income in Rwanda, with 1 being the lowest economic level and 4 being the highest

### Comprehensibility (semantic equivalence)

In focus group discussions and interviews, participants evaluated the Kinyarwanda terminology used to describe each item. When participants identified terms that were difficult to understand, alternative phrasing was developed. For example, in item #3 (“I feel all alone”), participants answered with specific people in mind who had or had not abandoned them. To address this, language was added to describe a more general feeling of abandonment. Because of the importance of religious faith in Rwandan culture, items #5 (“faith that gives comfort”) and #8 (“deep inner strength”) originally had similar meanings when directly translated into Kinyarwanda. To differentiate the two items, both items were reworded and subsequently correctly interpreted by participants and accurately back-translated. Similarly, some participants asked for clarification about the difference between item #2 (“goals”) and item #10 (“sense of direction”), but ultimately the majority of participants deemed the questions sufficiently distinct. With the initial translation of item #7 (“recall joyful times”), participants interpreted the question as asking them to compare present times to past times, so the language was adjusted to focus on the ability to remember joyful memories. The original translation of item #9 (“give and receive care and love”) elicited responses about whether or not participants had specific people to love currently in their lives, as opposed to having a general capacity to love and to be loved. The Kinyarwanda phrasing was changed to address this. See Additional file 1 for the final HHI-K and Additional file 2 for the English back-translation.

### Acceptability and other response set issues (technical equivalence)

**Fig. 1:**
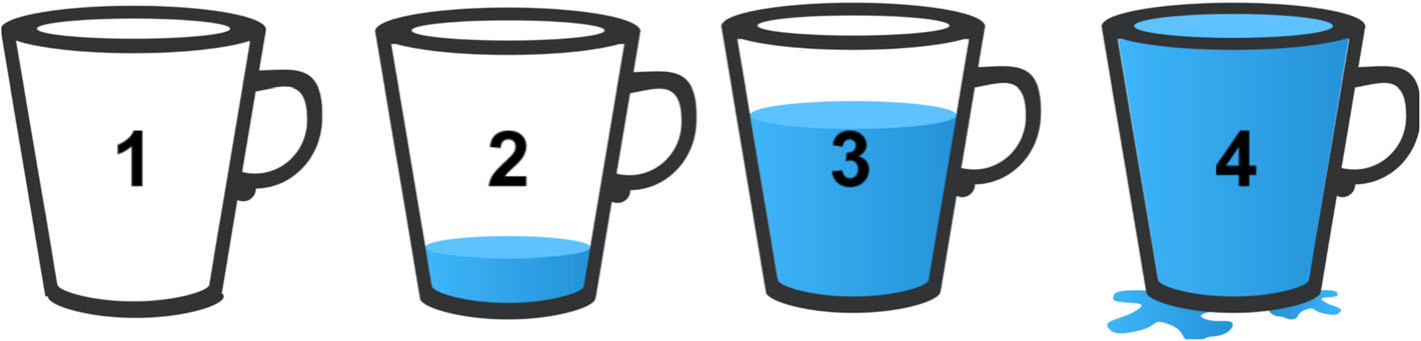
Visual response set of HHI-K

In the first step of the translation, the structure of the response set was revised. In the original English HHI, the response options are “strongly disagree,” “disagree,” “agree,” and “strongly agree,” correlating to scores of 1, 2, 3, and 4 respectively. Because of the varying education levels of the target population, translators deemed the Likert scale format difficult to interpret for participants when distinguishing between response options. As described in the cross-cultural mental health literature, technical equivalence is achieved when “the method of assessment … is comparable in each culture with respect to the data it yields.” [57] In Nepal and Afghanistan, water glasses filled to different levels have been used to represent response categories [58, 59]. Translators built on these previous adaptations to create a new visual response set featuring water cups (Fig. 1). Participants’ interpretations of the visual response set were comparable to the meaning of the data obtained through the original Likert scale.

Because of the varying literacy levels of the target population, questions were adapted to be asked verbally by survey enumerators, as opposed to being completed by participants on paper. This made declarative phrases difficult to understand. For example, enumerators reading item #1–“I have a positive outlook towards life”–confused respondents. Therefore, each HHI item was rephrased as a question: “Do you have a positive outlook towards life?” Additionally, during focus groups, participants expressed that answering on a scale from 1 to 4 in response to a yes or no question made it difficult to distinguish among response options. However, participants also found it confusing to immediately respond to “To what extent do you have a positive outlook towards life?” Participants explained that an initial yes or no question, followed by a second question that clarified to what degree the sentiment was endorsed, would be easiest to interpret. Each survey item was rephrased to include both questions. Questions were also modified to match the visual response set. For example, item #1 became “Do you believe that your life will be better in the future? Compare your belief with the cup illustration to indicate which cup best fits your answer choice.”

During focus group discussions, participants identified the order of the questions as a barrier to understanding. Item #3 (“I feel all alone”) and #6 (“I feel scared about my future”) are reverse scored, meaning that “strongly agree” corresponds to a score of 1. Because a full cup implies the most positive, or hopeful, response for all other items, focus group participants still considered the full cup to be positive when answering the reverse score questions. To address this, items #3 and #6 were moved to the end of the survey and became items #11 and #12, with additional explanations added to each. For example, after asking item #11, survey enumerators add, “Note: This question is different from the other questions. The full cup means that you have been abandoned and no one cares about you (4), whereas the empty cup means you have not been abandoned at all and you are generally cared for (1).” After adding these notes, participants interpreted and responded to the reverse scored questions correctly.

### Relevance and completeness (content and conceptual equivalence)

In the focus group discussions and interviews, all items were deemed relevant to the Rwandan context. Item #2 originally asked if respondents have “short and/or long-range goals,” but the difference between short and long-term goals was not directly translatable into Kinyarwanda. To keep the item as clear as possible, the final Kinyarwanda item #2 asks only about general goals without the specificity of short-term or long-term.

After revisions based on the results of the focus groups and interviews, the HHI-K underwent blind back-translation in which two fluent English and Kinyarwanda speakers unfamiliar with the HHI translated the HHI-K back to English. Minor edits were made after the black-translations and the tool was finalized for use in the psychometric evaluation.

### Psychometric properties

The HHI-K was administered to a sample of 206 participants (Table 2). For CFA, 10 participants per variable (including latent variables) are generally considered sufficient [60]. In this study, there were 15 variables, including the 12 indicator (observed) variables and 3 latent (unobserved) variables tested in CFA.

**Table 2:** Demographics of psychometric evaluation sample

Role in Health Center	Total Sample (***n*** = 206)
Healthcare Service User	180 (87%)
Healthcare Worker	26 (13%)
**Age, Mean (Range)**	32 (19–59)
**Gender**	
Male	5 (2%)
Female	201 (98%)
**Ubudehe Category**^a^	
1	22 (11%)
2	104 (50%)
3	78 (38%)
4	0 (0%)
Missing	2 (1%)
**Education**	
No schooling	34 (17%)
Primary	113 (55%)
Vocational Training School	14 (7%)
Secondary	26 (13%)
Advanced diploma	13 (6%)
Bachelor’s degree	5 (2%)
Missing	1 (0%)
**Marital Status**	
Single	55 (27%)
Legally Married	124 (60%)
Live-in/Not Legally Married	23 (11%)
Widowed	3 (1%)
Celibate / Religious	1 (0%)

^a^Ubudehe category refers to the economic classification of individuals based on their income in Rwanda, with 1 being the lowest economic level and 4 being the highest

The age of respondents ranged from 19 to 59 years old with a mean of 32. As in the translation and adaptation portion of the study, almost all participants were female (98%) and most were married or living with their partner (71%). The HHI-K had a mean of 37.87 out of 48 and a standard deviation of 6.17, slightly higher than the original HHI’s mean of 34.49 and standard deviation of 9.61 [23].

### Reliability

Cronbach’s alpha (CI 95%) for the HHI-K was 0.85 [0.82–0.88], demonstrating high internal consistency of the scale as a whole. The test-retest reliability coefficient (CI 95%) within 2 weeks was 0.85 [0.74–0.91] (ICC value). These results suggest that the HHI-K is a reliable instrument.

### Factor structure

**Fig. 2:**
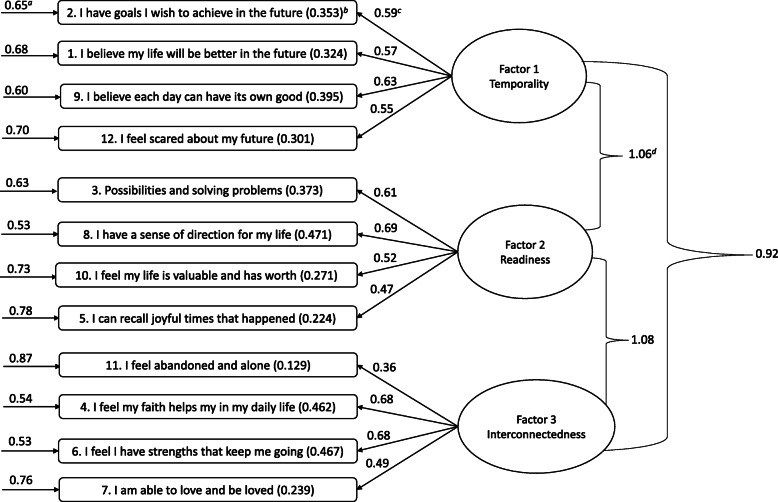
The Herth Hope Index-Kinyarwanda Measurement Model for the Three-Factor Model. a: Measurement Error. b: Squared Multiple Correlation (SMC) value. c: Standardized Factor Loadings. d: Latent Variable Covariance

The original HHI three-factor model was tested with CFA using a maximum likelihood estimation [23]. A correlation table is shown in Table 3, while standardized and unstandardized parameter estimates are provided in Table 4. Using five commonly reported fit statistics, CFA demonstrated that the three-factor model had adequate fitness indicators (Table 5). All indicator variable factor loadings were above 0.30, significant in the CFA literature [61, 62]. The squared multiple correlation (SMC) values are included in the three-factor model in Fig. 2 in italics. These values were used to determine the reliability of the latent variables because they represent the percentage of variance accounted for by a corresponding latent variable per indicator variable [61]. Across all indicator variables, the highest SMC was 0.471 (HHI-K8 for readiness) and the lowest was 0.129 (HHI-K11 for interconnectedness).

**Table 3:** Empirical Data Correlation Matrix

Observed variable	1	2	3	4	5	6	7	8	9	10	11	12
HHI 1	1	–	–	–	–	–	–	–	–	–	–	–
HHI 2	0.37	1	–	–	–	–	–	–	–	–	–	–
HHI 3	0.38	0.43	1	–	–	–	–	–	–	–	–	–
HHI 4	0.40	0.30	0.39	1	–	–	–	–	–	–	–	–
HHI 5	0.26	0.27	0.30	0.35	1	–	–	–	–	–	–	–
HHI 6	0.31	0.34	0.44	0.50	0.41	1	–	–	–	–	–	–
HHI 7	0.36	0.27	0.34	0.29	0.31	0.38	1	–	–	–	–	–
HHI 8	0.44	0.49	0.47	0.51	0.21	0.48	0.27	1	–	–	–	–
HHI 9	0.31	0.41	0.41	0.37	0.24	0.42	0.39	0.46	1	–	–	–
HHI 10	0.29	0.19	0.31	0.48	0.23	0.38	0.26	0.38	0.39	1	–	–
HHI 11	0.13	0.20	0.25	0.21	0.26	0.14	0.20	0.27	0.21	0.24	1	–
HHI 12	0.30	0.32	0.38	0.31	0.27	0.36	0.25	0.42	0.33	0.27	0.27	1

Correlation between raw survey scale score values using the Pearson correlation coefficient. *n* = 206. M = 0.39. SD = 0.2

**Table 4:** Standardized and Unstandardized Coefficients for CFA Analysis

Observed variable	Latent construct	β	B	SE
HHI 2	Temporality	0.59	1.00	
HHI 1	Temporality	0.57	1.10	0.17
HHI 9	Temporality	0.63	1.26	0.18
HHI 12	Temporality	0.55	1.22	0.19
HHI 3	Readiness	0.61	1.00	
HHI 8	Readiness	0.69	0.98	0.12
HHI 10	Readiness	0.521	0.65	0.10
HHI 5	Readiness	0.47	0.80	0.13
HHI 11	Interconnectedness	0.36	1.00	
HHI 4	Interconnectedness	0.68	1.72	0.36
HHI 6	Interconnectedness	0.68	1.89	0.40
HHI 7	Interconnectedness	0.49	1.36	0.32

*CFA* Confirmatory factor analysis. β: standardized coefficient. B: unstandardized coefficient. *SE* Standard error

**Table 5:** Fit statistics for confirmatory factor analysis of the HHI-K

Factor 1: Inner sense of temporality and future	Factor 2: Inner positive readiness and expectancy	Factor 3: Interconnectedness with self and others	X^**2**^/d.f.	X^**2**^	RMSEA	SRMR	CFI	TLI
1,2,9,12	3,5,8,10	4,6,7,11	1.53	77.89	0.05	0.05	0.96	0.95

X^2^/d.f.: normed chi square. X^2^: chi square. RMSEA: Root Mean Square Error of Approximation. SRMR: standardized root mean square residual. CFI: comparative fit index. TLI: Tucker-Lewis Index

## Discussion

The HHI-K demonstrates values of reliability similar to those shown in previous HHI studies [27, 63]. The original three-factor model of the HHI fits the HHI-K well. Other dimensionality and factor analysis models of HHI translations in the literature showed some instability across patient groups and cultures. Rustøen et al. reviewed the results of studies that utilized HHI factor analyses and found that in the 11 total studies that have examined the factor structure of the HHI in different languages, one, two, and three factor structures were identified [27]. These different results are likely due to the fact that hope as a construct varies in different samples, contexts, and countries, which should be further examined in different settings and analytic approaches.

This is the first study to conduct a cross-cultural validation of the Herth Hope Index to Kinyarwanda. The HHI-K showed satisfactory psychometric properties, with adequate reliability, as demonstrated by satisfactory internal consistency and test-retest reliability, and construct validity, as demonstrated by factor analysis. As the first published translation and evaluation of the HHI in an LMIC setting, the study’s results also illustrate that the construct of hope as measured by the HHI is relevant in various cultural contexts. With overall adequate psychometric properties, the HHI-K is a useful tool for research and program development in Rwandan healthcare settings. While there are many existing examples of cross-cultural translation and adaptation survey processes, the HHI-K is unique in that it provides a feasible model for adaptation of a positive tool for both patients and providers in low-resource settings. Further, building on the growing evidence base of the importance of hope to poverty alleviation, the HHI-K can be used to assess the effectiveness of programs that aim to promote hope and health outcomes at the health system-level, such as continuous quality improvement [64], ongoing training [65], mHealth [66], and improved inventory management [67], and at the individual level, such as peer support groups [68], stress management, motivational interviewing [69], mindfulness interventions [70], and cognitive-behavioral skills [71]. By using the HHI-K, health interventions can better understand and address hope and its role in provider burnout, quality of care, treatment uptake, and other program outcomes, ultimately helping to make aspirations towards UHC more attainable.

It is important to recognize that hope is not a cure-all for health disparities, and instilling hope without resources is unlikely to improve related health outcomes. However, there is promising research that health and poverty-alleviation programs can amplify their impact by showing people that a better life is attainable [6, 7], and the HHI-K is a valid tool to be used as part of such work.

### Limitations

Limitations related to the study design should be taken into account. This study used a purposive sample size to include participants represented in current TIP programs in Gakenke District, Rwanda, which are composed of predominantly female patients and providers. The nonprobability sample provides a limited perspective within the general population in Rwanda and thus the results presented should be considered within this scope; the survey might need to be adjusted for other Rwandan settings. Further, response bias could have influenced the results. Since the HHI-K is verbally enumerated, participants may have inflated their answers due to social desirability bias, meaning that participants answered sensitive questions with socially desirable, rather than truthful, answers. This risk was addressed by ensuring that participants did not know survey enumerators before answering the survey and that the survey was administered in private spaces. If participants asked clarifying questions about the meaning of survey items, enumerators were trained to reread each item so that responses would not be biased due to potentially different explanations from enumerators.

## Conclusion

The HHI-K is a reliable and valid instrument to measure hope in Rwanda. The study also demonstrates that it is feasible to cross-culturally adapt and validate a positive psychosocial measure for healthcare workers and recipients, and the process used can serve as a model for future adaptations in low-resource settings. Given its satisfactory psychometric properties, the HHI-K can be used to provide relevant information to support global health practitioners in understanding the role of hope among healthcare patients and providers in Rwanda, aiding evaluations of relevant health programs. Further studies should conduct adaptations and evaluations of the HHI in other samples and contexts to further confirm the factor stability found in the results. This initial report on the psychometric properties of the HHI-K should be used to generate further research on the capacity to measure and influence hope in LMICs, allowing for more evidence-based programs to influence hope and improve health going forward.

## Supplementary information

**Additional file 1.** Herth Hope Index-Kinyarwanda.

**Additional file 2.** English back-translation of the Herth Hope Index-Kinyarwanda.

## Data Availability

The datasets used and/or analyzed during the current study are available from the corresponding author on reasonable request.

## Abbreviations

CFA: Confirmatory factor analysis
CFI: Comparative fix index
LMIC: Low- and middle-income country
HHI-K: Herth Hope Index-Kinyarwanda
ICC: Intraclass correlation coefficient
RMSEA: Root mean square error of approximation
SD: Standard deviation
SE: Standard error
SMC: Squared multiple correlation
SRMR: Standardized root mean square residual
TIP: TIP Global Health
TLI: Tucker-Lewis Index
UHC: Universal health coverage
