# Supplementary material for: Cross-cultural adaptation and psychometric properties of the Herth Hope Index in Kinyarwanda: adapting a positive psychosocial tool for healthcare recipients and providers in the Rwandan setting
Source: Health Qual Life Outcomes. 2020 Aug 24;18:286. doi: 10.1186/s12955-020-01537-3 (PMC7444040; doi:10.1186/s12955-020-01537-3)
Supplement: Supplementary file 1 — Additional file 1. Herth Hope Index-Kinyarwanda. [file 12955_2020_1537_MOESM1_ESM.pdf]

Additional file 1. Herth Hope Index-Kinyarwanda

Amabwiriza:

Turaza kubabaza ibibazo cumi na bibiri namwe musubize muri rusange uko mubyumva. Muraza gusubiza mbere yego cyangwa oya, mukurikije uko ikibazo kibaza. Murakomeza gusobanura icyo gisubizo cyanyu mukurikije uko mwemera/mwumva ikibazo mugendeye/mwifashishije ibikombe kuva ku gikombe kirimo ubusa kugera ku gikombe cyuzuye/Gisendereye. Igikombe kirimo ubusa aricyo 1 ni igihe utemera na gato ikibazo cyangwa bitajya bikubaho na gato, naho igikombe cyuzuye aricyo 4 ni igihe wemera cyane ikibazo cyangwa bijya bikubaho kenshi.

Ubaza aha urupapuro ubazwa ruriho ibikombe akaba arufite mu ntoki ze, bityo ubazwa akaba yakwihitiramo igisubizo kuri buri kibazo yifashishije urwo rupapuro ruriho ibikombe.

	**1**	**2**	**3**	**4**
1. Wumva wizeye ko ubuzima bwawe cyangwa imibereho yawe bizaba byiza kurushaho mu bihe biri imbere? Gereranya uko icyizere cyawe kingana wifashishije kimwe muri ibi bikombe.				
2. Wumva hari intego uteganya kugeraho mu bihe biri imbere? Gereranya intego ufite na kimwe muri ibi bikombe.				
3. Ujya wumva ko ushobora kubona inzira zo kuva mu bibazo mu gihe uhuye nabyo? Gereranya uburyo ubona inzira zo kuva mu bibazo uhura nabyo wifashishije kimwe muri ibi bikombe.				

	1	2	3	4
4. Ese ukwemera kwawe wumva hari icyo kugufasha mubuzima bwa buri munsi? Gereranya uko wumva ukwemera kwawe kugufasha mu buzima bwa buri munsi wifashishije kimwe muri ibi bikombe.				
5. Ese ujya wibuka ibihe bishimishije byakubayeho? Gereranya uburyo wibuka ibihe bishimishije byakubayeho wifashishije kimwe muri ibi bikombe.				
6. Wumva wifitemo imbaraga zigutera gukomera mu buzima bwa buri munsi? Gereranya imbaraga wifitemo na kimwe muri ibi bikombe.				
7. Muri wowe wumva, ukunda kandi ukunzwe? Gereranya uburyo ukunda kandi wumva ukunzwe ukoresheje kimwe muri ibi bikombe.				
8. Ese waba ufite icyerekezo cy'aho ushaka kugera mu buzima bwawe? Gereranya uko kugira icyerekezo kwawe na kimwe muri ibi bikombe.				
9. Ese wemera ko umunsi uwo ari wo wose ugira ibyiza byawo? Gereranya uko kwemera kwawe na kinwe muri ibi bikombe.				

	1	2	3	4
10. Ese wumva ubuzima bwawe bufite agaciro kandi bugakwiriye? Gereranya uko wumwa agaciro k'ubuzima bwawe na kimwe muri ibi bikombe.				
11. Ubona muri rusange abantu baragutereranye ku buryo ubona nta n'umwe ukwitayeho? Gereranya ugutereranwa kwawe na kimwe muri ibi bikombe.<br><br>Icyitonderwa: Iki kibazo gitandukanye n'ibindi bibazo. Agakombe kuzuye gasobanura ko umuntu yatereranywe cyane kandi ko nta numwitayeho, naho agakombe karimo ubusa, gasobanura ko utatereranye na gato kandi ko witaweho muri rusange.				
12. Ujya wumva utewe ubwoba n'ejo hazaza hawe? Gereranya ubwoba ugirira ejo hazaza na kimwe muri ibi bikombe.<br><br>Icyitonderwa: Iki kibazo gitandukanye n'ibindi bibazo. Agakombe kuzuye gasobanura ko ufite ubwoba bwinshi bw'ejo hazaza hawe, naho agakombe karimo ubusa, gasobanura ko nta bwoba bw'ejo hazaza ufite.				

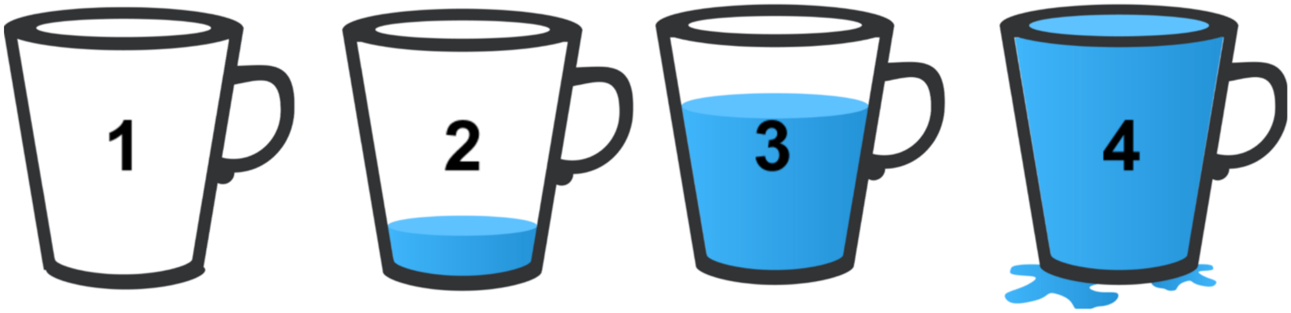
1
2
3
4
