# Supplementary material for: Cross-cultural adaptation and psychometric properties of the Herth Hope Index in Kinyarwanda: adapting a positive psychosocial tool for healthcare recipients and providers in the Rwandan setting
Source: Health Qual Life Outcomes. 2020 Aug 24;18:286. doi: 10.1186/s12955-020-01537-3 (PMC7444040; doi:10.1186/s12955-020-01537-3)
Supplement: Supplementary file 2 — Additional file 2. English back-translation of the Herth Hope Index-Kinyarwanda. [file 12955_2020_1537_MOESM2_ESM.pdf]

Additional file 2. English back-translation of the Herth Hope Index-Kinyarwanda

Instructions

We will ask you 12 questions and you will answer them based on your general understanding. You will first answer yes or no based on the question. You will continue to explain your answer based on how you agree or disagree with the question using the help of the cups, from the empty cup to the full cup. The empty cup, which is cup 1, is for when you do not agree with the question at all or if it never happens to you, and the full cup, which is cup 4, is for when you agree very much with the question or if it happens to you often.

The surveyor gives a handout with the cup illustration to the respondent for them to keep at hand. The respondent will independently choose an answer to each question using the handout with the cup illustration.

1. Do you believe that your life or your livelihood will be better in the future? Compare your belief with the cup illustration to indicate which cup best fits your answer choice.

2. Do you have goals that you wish to achieve in the future? Compare the goals you have with the cup illustration to indicate which cup best fits your answer choice.

3. Do you feel like you have possibilities and ways of solving problems that you face in your life? Compare your belief that you can find possibilities when you face problems with the cup illustration to indicate which cup best fits your answer choice.

4. Do you feel that your faith helps you in your daily life? Compare the way that your faith helps you in your daily life with the cup illustration to indicate which cup best fits your answer choice.

5. Do you recall the joyful times that happened to you? Compare the way you remember the joyful times with the cup illustration to indicate which cup best fits your answer choice.

6. Do you feel that you have strength that keeps you going in your daily life? Compare your strength with the cup illustration to indicate which cup best fits your answer choice.

7. Do you feel able to love and to be loved? Compare how you are able to love and be loved with the cup illustration to indicate which cup best fits your answer choice.

8. Do you have a sense of direction of where you would like to be in your life? Compare your sense of direction with the cup illustration to indicate which cup best fits your answer choice.

9. Do you believe that each day can have its own good? Compare that belief with the cup illustration to indicate which cup best fits your answer choice.

10. Do you feel that your life is valuable and has worth? Compare the way you feel your life has value with the cup illustration to indicate which cup best fits your answer choice.

11. Do you generally feel that people have abandoned you and that nobody cares about you? Compare your feeling of abandonment with the cup illustration to indicate which cup best fits your answer choice.

> *Note*: This question is different from the other questions. The full cup (4) means that you have been abandoned and no one cares about you, whereas the empty cup (1) means you have not been abandoned at all and you are generally cared for.

12. Do you feel scared about your future? Compare the fear you have about your future with the cup illustration to indicate which cup best fits your answer choice.

> *Note*: This question is different from the other questions. The full cup (4) means that you have a lot of fear about your future, whereas an empty cup (1) means that you do not have any fear about your future.
